# Supplementary material for: Identification of Differentially Expressed Genes in Resistant Tetraploid Wheat (Triticum turgidum) under Sitobion avenae (F.) Infestation
Source: Int J Mol Sci. 2022 May 27;23(11):6012. doi: 10.3390/ijms23116012 (PMC9180832; doi:10.3390/ijms23116012)
Supplement: Supplementary file 1 [file ijms-23-06012-s001.zip › ijms-1718443-supplementary.pdf]

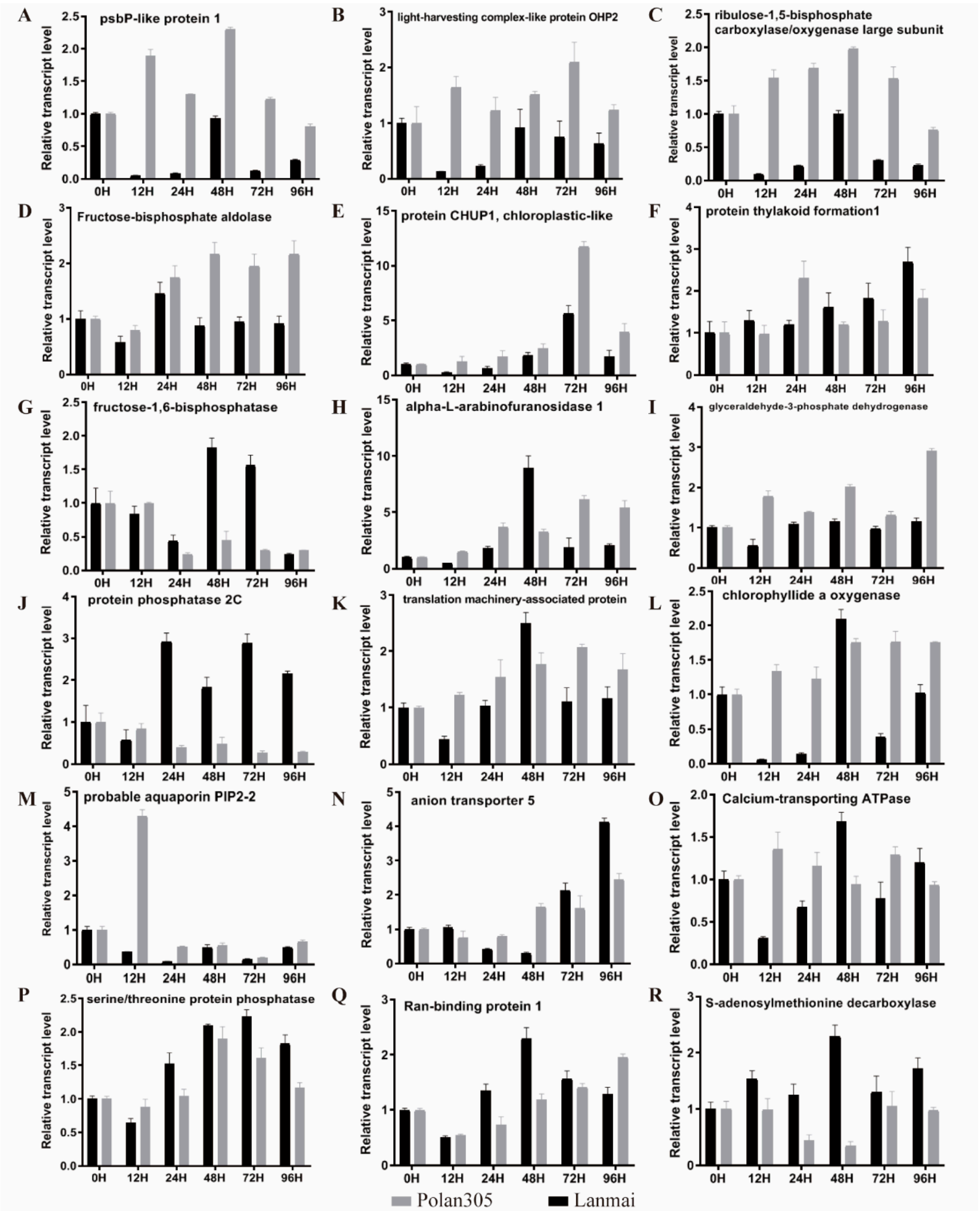

Supplementary Figure S1. Expression profiles of 18 representative ESTs in seedlings of the resistant (Lanmai) and susceptible (Polan305) varieties under *S. avenae* damage at different times. RT-qPCR was used to detect the expression levels of the candidate genes. *TaActin* was used as an internal control. Each value represents the mean ± SE of three biological replicates. psbP-like protein 1 **(A)**, light-harvesting complex-like protein OHP2 **(B)**, ribulose-1,5-bisphosphate carboxylase/oxygenase large subunit **(C)**, Fructose-bisphosphate aldolase **(D)**, protein CHUP1, chloroplastic-like**(E)**, protein thylakoid formation 1 **(F)**, fructose-1,6-bisphosphatase **(G)**, alpha-L-arabinofuranosidase 1 **(H)**, glyceraldehyde-3-phosphate dehydrogenase **(I)**, protein phosphatase 2C **(J)**, translation machinery-associated protein **(K)**, chlorophyllide, an oxygenase **(L)**, probable aquaporin TIP2-2 **(M)**, anion transporter 5 **(N)**, calcium-transporting ATPase 9 **(O)**, serine/threonine protein phosphatase **(P)**, RAN-binging protein 1 **(Q)**,S-adenosylmethionine decarboxylase **(R)**.

Supplementary Table S1. The list of ESTs of SSH cDNA library from tetraploid wheat infected by S. avenae feeding

dbEST_Id	User_Id	GenBank_Accn	Nr annnotation			wheat function annnotation		Mapman function annotation		Gene_Ontology
			Protein name/organism	E value	Per. Ident	gene ID	annnotation	BIN code	name	
Photosynthesis and energy										
75911204	S0049	JK716040	psbP-like protein 1, chloroplastic [Triticum aestivum]	5.00E-80	99.17%	TraesCS4A03G0778600	PsbP-like protein 1	1.1.1.2	PS.lightreaction.photosystem II.PSII polypeptide subunits	GO:0005509 MF: calcium ion binding;GO:0009523 CC: photosystem II;GO:0009654 CC: photosystem II oxygen evolving complex;GO:0015979 BP: photosynthesis;GO:0019898 CC: extrinsic component of membrane
75911207	S0113	JK716043	light-harvesting complex-like protein OHP2, chloroplastic [Triticum dicoccoides]	5.00E-29	100.00%	TraesCS2D03G1329300	Photosystem II CP43 reaction center protein	1.1.1.2	PS.lightreaction.photosystem II.PSII polypeptide subunits	GO:0009521 CC: photosystem;GO:0009767 BP: photosynthetic electron transport chain;GO:0016020 CC: membrane;GO:0016168 MF: chlorophyll binding;GO:0019684 BP: photosynthesis, light reaction
75911209	S0115	JK716045	protein curvature thylakoid 1B, chloroplastic-like [Triticum dicoccoides]	2.00E-07	100.00%	TraesCS1B03G0625000	Protein curvature thylakoid chloroplastic-like	1.1.2.2	PS.lightreaction.photosystem I.PSI polypeptide subunits	Unknown
75912217	S0223	JK716053	SKP1-like protein 1[Triticum dicoccoides]	3.00E-136	97.28%	TraesCS7D03G0036700	SKP1-like protein	1.1.5.3	PS.lightreaction.other electron carrier (ox/red).ferredoxin reductase	GO:0006511 BP: ubiquitin-dependent protein catabolic process
75912331	S0337	JK716067	photosystem I reaction center subunit N, chloroplastic-like [Triticum dicoccoides]	9.00E-49	97.47%	TraesCS2D03G0240900	Photosystem I reaction center subunit N	1.1.2.2	PS.lightreaction.photosystem I.PSI polypeptide subunits	GO:0009522 CC: photosystem I;GO:0015979 BP: photosynthesis

75911246	S052	JK716082	Chlorophyll a-b binding protein 1C, chloroplastic [Triticum urartu]	4.00E-27	100.00%	TraesCS1B03G0871200	Chlorophyll a-b binding protein, chloroplastic	1.1.1.1	PS.lightreaction.photosystem II.LHC-II	Unknown
75912547	S053	JK716083	Chlorophyll a-b binding protein 1C, chloroplastic [Triticum urartu]	7.00E-25	100.00%	TraesCS5B03G0884400	Chlorophyll a-b binding protein, chloroplastic	1.1.1.1	PS.lightreaction.photosystem II.LHC-II	Unknown
75912655	S061	JK716091	photosystem II 10 kDa polypeptide, chloroplastic [Triticum dicoccoides]	6.00E-55	89.21%	TraesCS6A03G0951000	Photosystem II 10 kDa polypeptide family protein	1.1.1.2	PS.lightreaction.photosystem II.PSII polypeptide subunits	GO:0009523 CC: photosystem II;GO:0009654 CC: photosystem II oxygen evolving complex;GO:0015979 BP: photosynthesis;GO:0042651 CC: thylakoid membrane
75912663	S069	JK716099	protein THYLAKOID FORMATION1, chloroplastic [Triticum dicoccoides]	6.00E-23	100.00%	TraesCS2B03G0560400	Protein THYLAKOID FORMATION1, chloroplastic	1.1.1.2	PS.lightreaction.photosystem II.PSII polypeptide subunits	GO:0010207 BP: photosystem II assembly;GO:0015979 BP: photosynthesis
75911312	S118	JK716148	vulgare photosystem II repair protein PSB27-H1, chloroplastic[Hordeum vulgare]	7.00E-152	99.02%	TraesCS4D03G0429100	Photosystem II Psb27 protein	1.1.1.2	PS.lightreaction.photosystem II.PSII polypeptide subunits	GO:0010207 BP: photosystem II assembly
75911329	S001	JK716165	ribulose-1,5-bisphosphate carboxylase/oxygenase large subunit [Triticum aestivum]	0	97.86%	TraesCS3B03G0446200	Ribulose bisphosphate carboxylase large chain	1.3.1	PS.calvin cycle.rubisco large subunit	GO:0000287 MF: magnesium ion binding;GO:0015977 BP: carbon fixation;GO:0016984 MF: ribulose-bisphosphate carboxylase activity
75911097	S002	JK716033	ribulose-1,5-bisphosphate carboxylase/oxygenase large subunit [Hordeum brachyantherum]	2.00E-47	100.00%	TraesCS5D03G0016000	Ribulose bisphosphate carboxylase large chain	1.3.1	PS.calvin cycle.rubisco large subunit	GO:0000287 MF: magnesium ion binding;GO:0015977 BP: carbon fixation;GO:0016984 MF: ribulose-bisphosphate carboxylase activity

75911098	S003	JK716034	ribulose bisphosphate carboxylase large chain-like [Triticum aestivum]	7.00E-63	99.02%	TraesCS5D03G0936400	Ribulose bisphosphate carboxylase large chain	1.3.1	PS.calvin cycle.rubisco large subunit	GO:0000287 MF: magnesium ion binding;GO:0015977 BP: carbon fixation;GO:0016984 MF: ribulose-bisphosphate carboxylase activity
75911099	S004	JK716035	ribulose 1,5-bisphosphate carboxylase/oxygenase large subunit, Rubisco [Triticum aestivum]	4.00E-11	93.75%	TraesCS5D03G0936400	Ribulose bisphosphate carboxylase large chain	1.3.1	PS.calvin cycle.rubisco large subunit	GO:0000287 MF: magnesium ion binding;GO:0015977 BP: carbon fixation;GO:0016984 MF: ribulose-bisphosphate carboxylase activity
75911202	S007	JK716038	ribulose bisphosphate carboxylase small chain PW9, chloroplastic-like [Triticum dicoccoides]	1.00E-109	91.43%	TraesCS5D03G0414200	Ribulose bisphosphate carboxylase small chain	1.3.2	PS.calvin cycle.rubisco small subunit	Unknown
75911203	S008	JK716039	ribulose bisphosphate carboxylase small chain PW9, chloroplastic-like [Triticum dicoccoides]	5.00E-43	98.53%	TraesCS2A03G0131800	Ribulose bisphosphate carboxylase small chain	1.3.2	PS.calvin cycle.rubisco small subunit	Unknown
75911330	S011	JK716166	ribulose bisphosphate carboxylase/oxygenase activase A, chloroplastic-like isoform X1 [Triticum dicoccoides]	1.00E-64	96.84%	TraesCS4B03G0327500	Ribulose bisphosphate carboxylase/oxygenase activase	1.3.13	PS.calvin cycle.rubisco interacting	GO:0005524 MF: ATP binding
75911230	S036	JK716066	ribulose-1,5-bisphosphate carboxylase/oxygenase large subunit [Triticum turgidum]	4.000E-75	100.00%	TraesCS3B03G0446200	Ribulose bisphosphate carboxylase large chain	1.3.1	PS.calvin cycle.rubisco large subunit	GO:0000287 MF: magnesium ion binding;GO:0015977 BP: carbon fixation;GO:0016984 MF: ribulose-bisphosphate carboxylase activity
75911240	S046	JK716076	fructose-1,6-bisphosphatase, cytosolic-like [Triticum dicoccoides]	3.00E-119	94.41%	TraesCS3D03G0819500	Fructose-1,6-bisphosphatase class 1	1.3.7	PS.calvin cycle.FBPase	GO:0005975 BP: carbohydrate metabolic process;GO:0016791 MF: phosphatase activity;GO:0042132 MF: fructose 1,6-bisphosphate 1-phosphatase activity
75911273	S079	JK716109	phospholipase A1-II 7-like [Triticum dicoccoides]	2.00E-65	96.6	TraesCS4D03G0	Fructose-bisphosphate aldolase	1.3.6	PS.calvin cycle.aldolase	GO:0003824 MF: catalytic activity;GO:0004332 MF: fructose-bisphosphate aldolase activity;GO:0006096 BP: glycolytic process

					4%	211900				
Cell organisation and division										
75911213	S019	JK716049	cell division control protein 48 homolog E-like [Triticum dicoccoides]	6.00E-25	97.26%	TraesCS4B03G0713200	Cell division cycle protein 48-like protein	31.2	cell.division	GO:0005524 MF: ATP binding;GO:0016787 MF: hydrolase activity
75911244	S050	JK716080	protein CHUP1, chloroplastic-like [Triticum dicoccoides]	6.00E-92	97.26%	TraesCS1B03G1195400	Protein CHUP1, chloroplastic	31.1	cell.organisation	GO:0005179 MF: hormone activity;GO:0005576 CC: extracellular region
75911245	S051	JK716081	protein CHUP1, chloroplastic-like [Triticum dicoccoides]	3.00E-63	98.13%	TraesCS1A03G1014100	Protein CHUP1, chloroplastic	31.1	cell.organisation	GO:0005179 MF: hormone activity;GO:0005576 CC: extracellular region
75911284	S090	JK716120	MOB kinase activator-like 1A [Hordeum vulgare]	6.00E-60	97.96%	TraesCS6D03G0369700	MOB kinase activator-like 1A	31.2	cell.division	Unknown
Cell wall										
75911265	S071	JK716101	probable aquaporin TIP2-2 [Triticum dicoccoides]	4.00E-90	86.24%	TraesCS7A03G0816900	Aquaporin	10.7	cell wall.modification	GO:0005215 MF: transporter activity;GO:0006810 BP: transport;GO:0016020 CC: membrane
75911290	S096	JK716126	expansin-B6-like [Triticum dicoccoides]	1.00E-43	94.87%	TraesCS1B03G0651300	Expansin protein	10.7	cell wall.modification	GO:0005576 CC: extracellular region;GO:0019953 BP: sexual reproduction

75911315	S121	JK716151	probable aquaporin PIP2-2 [Triticum dicoccoides]	3.00E-85	87.99%	TraesCS4B03G0037300	Bifunctional inhibitor/lipid-transfer protein/seed storage 2S albumin superfamily protein	35.2	not assigned.unknown	Unknown
Metabolism										
75911201	S006	JK716037	4-hydroxyphenylpyruvate dioxygenase-like [Triticum dicoccoides]	5.00E-05	92.86%	TraesCS6B03G0433700	4-hydroxyphenylpyruvate dioxygenase	12.4	N-metabolism.misc	GO:0003868 MF: 4-hydroxyphenylpyruvate dioxygenase activity;GO:0009072 BP: aromatic amino acid family metabolic process;GO:0016701 MF: oxidoreductase activity, acting on single donors with incorporation of molecular oxygen;GO:0055114 BP: oxidation-reduction process
75911221	S027	JK716057	glucose-6-phosphate isomerase 1, chloroplastic-like [Triticum dicoccoides]	2.00E-58	84.13%	TraesCS5D03G0584200	Glucose-6-phosphate isomerase	4.2.3	glycolysis.plastid branch.glucose-6-phosphate isomerase	GO:0004347 MF: glucose-6-phosphate isomerase activity;GO:0006094 BP: gluconeogenesis;GO:0006096 BP: glycolytic process
75911222	S028	JK716058	photosystem II 22 kDa protein 1, chloroplastic-like [Triticum dicoccoides]	5.00E-46	95.24%	TraesCS6B03G0884100	AT-rich interactive domain protein	13.1.4.1.1	amino acid metabolism.synthesis.branched chain group.common.acetolactate synthase	GO:0003677 MF: DNA binding
75911224	S030	JK716060	malate dehydrogenase, cytoplasmic-like [Triticum dicoccoides]	1.00E-99	96.08%	TraesCS1B03G0509100	Malate dehydrogenase	8.2.9	TCA / org. transformation.other organic acid transformaitons.cyt MDH	GO:0003824 MF: catalytic activity;GO:0005975 BP: carbohydrate metabolic process;GO:0006108 BP: malate metabolic process;GO:0016491 MF: oxidoreductase activity;GO:0016615 MF: malate dehydrogenase activity;GO:0016616 MF: oxidoreductase activity, acting on the CH-OH group of donors, NAD or NADP as acceptor;GO:0019752 BP: carboxylic acid metabolic process;GO:0030060 MF: L-malate dehydrogenase activity;GO:0055114 BP: oxidation-reduction process
75911232	S038	JK716068	glyceraldehyde-3-phosphate dehydrogenase 1, cytosolic-like [Triticum dicoccoides]	1.00E-52	95.74%	TraesCS7D03G0737500	Glyceraldehyde-3-phosphate dehydrogenase	4.2.3	glycolysis.plastid branch.Glyceraldehyde 3-phosphate dehydrogenase	GO:0006006 BP: glucose metabolic process;GO:0016620 MF: oxidoreductase activity, acting on the aldehyde or oxo group of donors, NAD or NADP as acceptor;GO:0050661 MF: NADP binding;GO:0051287 MF: NAD binding;GO:0055114 BP: oxidation-reduction process
75911241	S047	JK716077	ATP synthase CF1 alpha subunit [Triticum turgidum]	4.00E-48	95.51%	TraesCS5D03G1016000	ATP synthase subunit alpha	9.9	mitochondrial electron transport / ATP synthesis.F1-ATPase	GO:0005524 MF: ATP binding;GO:0015986 BP: ATP synthesis coupled proton transport;GO:0015992 BP: proton transport;GO:0045261 CC: proton-transporting ATP synthase complex, catalytic core F(1);GO:0046034 BP: ATP metabolic process;GO:0046933 MF: proton-transporting ATP synthase activity, rotational mechanism

75911242	S048	JK716078	carbonic anhydrase, chloroplastic-like isoform X1 [Triticum dicoccoides]	3.00E-80	96.75%	TraesCS3A03G0593600	Carbonic anhydrase	8.3	TCA / org. transformation.carbonic anhydrases	GO:0004089 MF: carbonate dehydratase activity;GO:0008270 MF: zinc ion binding;GO:0015976 BP: carbon utilization
75911261	S067	JK716097	vulgare mRNA for predicted protein [Hordeum vulgare]	6.00E-168	99.11%	TraesCS6D03G0318800	Aldose 1-epimerase family protein			GO:0003824 MF: catalytic activity;GO:0005975 BP: carbohydrate metabolic process;GO:0016853 MF: isomerase activity;GO:0030246 MF: carbohydrate binding
75911271	S077	JK716107	kinetochore-associated protein KNL-2 homolog [Triticum turgidum]	9.00E-105	96.61%	TraesCS2A03G0682200	Acetyl-coenzyme A carboxylase carboxyl transferase subunit alpha			Unknown
75911277	S083	JK716113	malate dehydrogenase [NADP] 1, chloroplastic-like [Triticum dicoccoides]	2.00E-164	99.12%	TraesCS7B03G0546000	Malate dehydrogenase	35.2	not assigned.unknown	GO:0003824 MF: catalytic activity;GO:0005975 BP: carbohydrate metabolic process;GO:0006108 BP: malate metabolic process;GO:0016491 MF: oxidoreductase activity;GO:0016615 MF: malate dehydrogenase activity;GO:0016616 MF: oxidoreductase activity, acting on the CH-OH group of donors, NAD or NADP as acceptor;GO:0046554 MF: malate dehydrogenase (NADP+) activity;GO:0055114 BP: oxidation-reduction process
75911280	S086	JK716116	carbamoyl-phosphate synthase small chain, chloroplastic-like [Triticum dicoccoides]	9.00E-123	97.21%	TraesCS5D03G0395400	Carbamoyl-phosphate synthase small chain	23.1.1.1	nucleotide metabolism.synthesis.pyrimidine.carbamoyl phosphate synthetase	GO:0004088 MF: carbamoyl-phosphate synthase (glutamine-hydrolyzing) activity;GO:0006207 BP: 'de novo' pyrimidine nucleobase biosynthetic process;GO:0006541 BP: glutamine metabolic process
75911287	S093	JK716123	adenosylhomocysteinase [Triticum dicoccoides]	2.00E-117	98.84%	TraesCS2B03G1307900	Adenosylhomocysteinase	13.1.4.1.1	amino acid metabolism.synthesis.branched chain group.common.acetolactate synthase	GO:0004013 MF: adenosylhomocysteinase activity;GO:0006730 BP: one-carbon metabolic process;GO:0019510 BP: S-adenosylhomocysteine catabolic process;GO:0051287 MF: NAD binding
75911294	S100	JK716130	alpha-L-arabinofuranosidase 1-like isoform X2 [Triticum dicoccoides]	9.00E-101	99.32%	TraesCS4B03G0301000	Alpha-L-arabinofuranosidase 1	35.2	not assigned.unknown	GO:0046373 BP: L-arabinose metabolic process;GO:0046556 MF: alpha-L-arabinofuranosidase activity
75911297	S103	JK716133	sucrose synthase 2-like [Triticum dicoccoides]	4.00E-10	93.94%	TraesCS4A03G1116300	Sucrose synthase	2.2.1.5	major CHO metabolism.degradation.sucrose.Susy	GO:0005985 BP: sucrose metabolic process;GO:0016157 MF: sucrose synthase activity

75911301	S107	JK716137	alpha-L-arabinofuranosidase 1-like isoform X2 [Triticum dicoccoides]	1.00E-52	91.43%	TraesCS4A03G0506800	Alpha-L-arabinofuranosidase 1	35.2	not assigned.unknown	GO:0046373 BP: L-arabinose metabolic process;GO:0046556 MF: alpha-L-arabinofuranosidase activit
75911306	S112	JK716142	formamidase-like [Triticum dicoccoides]	2.00E-79	99.18%	TraesCS3A03G0747100	Acetamidase/formamidase, putative	26.1	misc.misc2	GO:0016811 MF: hydrolase activity, acting on carbon-nitrogen (but not peptide) bonds, in linear amides
Protein degradation										
75911205	S010	JK716041	ATP-dependent Clp protease ATP-binding subunit clpA-like protein CD4B, chloroplastic [Triticum urartu]	1.00E-27	96.61%	TraesCS5B03G0299500	ATP-dependent Clp protease ATP-binding subunit	29.5.5	protein.degradation.serine protease	GO:0005515 MF: protein binding;GO:0005524 MF: ATP binding;GO:0019538 BP: protein metabolic process
75911228	S034	JK716064	ATP-dependent Clp protease proteolytic subunit 3, chloroplastic-like [Triticum dicoccoides]	7.00E-161	98.22%	TraesCS3A03G0499900	ATP-dependent Clp protease proteolytic subunit	29.5.5	protein.degradation.serine protease	GO:0004252 MF: serine-type endopeptidase activity;GO:0006508 BP: proteolysis
75911248	S054	JK716084	protein SUPPRESSOR OF K(+) TRANSPORT GROWTH DEFECT 1 [Triticum dicoccoides]	3.00E-22	95.83%	TraesCS3A03G0112700	Katanin p60 ATPase-containing subunit, putative	29.5.9	protein.degradation.AAA type	GO:0005524 MF: ATP binding
75911252	S058	JK716088	protein HHL1, chloroplastic-like [Triticum dicoccoides]	3.00E-62	99.02%	TraesCS6B03G0712700	Protein HHL1, chloroplastic	29.5.11.20	protein.degradation.ubiquitin.proteasom	Unknown
75911276	S082	JK716112	20S proteasome beta 4 subunit [Triticum aestivum]	4.00E-23	95.83%	TraesCS4B03G0078000	Proteasome subunit beta type	29.5.11.20	protein.degradation.ubiquitin.proteasom	GO:0004175 MF: endopeptidase activity;GO:0004298 MF: threonine-type endopeptidase activity;GO:0005839 CC: proteasome core complex;GO:0051603 BP: proteolysis involved in cellular protein catabolic process

75 91 12 985	S 0 9 1	JK7 161 21	coiled-coil domain-containing protein 12-like [Triticum dicoccoides]	1.00E-61	94.34%	TraesCS7A03G1372300	Coiled-coil domain-containing protein 12	29.5	protein.degradation	Unknown
75 91 12 986	S 0 9 2	JK7 161 22	ubiquitin carboxyl-terminal hydrolase 12-like isoform X1 [Triticum dicoccoides]	2.00E-90	99.29%	TraesCS5A03G0275200	Ubiquitin carboxyl-terminal hydrolase, putative	29.5.11.5	protein.degradation.ubiquitin.ubiquitin protease	GO:0005515 MF: protein binding;GO:0006511 BP: ubiquitin-dependent protein catabolic process;GO:0016579 BP: protein deubiquitination;GO:0036459 MF: thiol-dependent ubiquitinyl hydrolase activity
75 91 13 000	S 1 0 6	JK7 161 36	E3 ubiquitin-protein ligase RNF25-like [Triticum dicoccoides]	3.00E-49	100.00%	TraesCS4B03G0407800	E3 ubiquitin-protein ligase RNF25	29.5.11.4.2	protein.degradation.ubiquitin.E3.RING	GO:0005515 MF: protein binding;GO:0008270 MF: zinc ion binding
75 91 13 104	S 1 1 0	JK7 161 40	E3 ubiquitin-protein ligase At1g63170-like isoform X1 [Triticum dicoccoides]	3.00E-59	98.94%	TraesCS5A03G0484600	RING/U-box superfamily protein	29.5.11.4.2	protein.degradation.ubiquitin.E3.RING	O:0005515 MF: protein binding;GO:0008270 MF: zinc ion binding
Protein synthesis										
75 91 12 184	S 0 2 4	JK7 160 54	40S ribosomal protein S15-like [Triticum dicoccoides]	5.00E-130	100.00%	TraesCS5A03G1045300	30S ribosomal protein S19	29.2.1.2.1.15	protein.synthesis.ribosomal protein.eukaryotic.40S subunit.S15	GO:0003723 MF: RNA binding;GO:0003735 MF: structural constituent of ribosome;GO:0005840 CC: ribosome;GO:0006412 BP: translation;GO:0015935 CC: small ribosomal subunit
75 91 12 206	S 0 2 6	JK7 160 56	vulgare mRNA for predicted protein[Hordeum vulgare]	2.00E-65	98.64%	TraesCS7B03G0291000	Receptor-like protein kinase			GO:0004672 MF: protein kinase activity;GO:0005515 MF: protein binding;GO:0005524 MF: ATP binding;GO:0006468 BP: protein phosphorylation
75 91 12 239	S 0 2 9	JK7 160 59	protein SGT1 homolog [Triticum dicoccoides]	1.00E-54	100.0	TraesCS3D03G0539200	SGT1	29.4	protein.postranslational modification	GO:0005515 MF: protein binding

					0%					
75911227	S033	JK716063	CTD small phosphatase-like protein 2 [Triticum dicoccoides]	5.00E-110	96.13%	TraesCS5B03G1308800	CTD small phosphatase-like protein	26.13	misc.acid and other phosphatases	GO:0016791 MF: phosphatase activity
75912436	S042	JK716072	neurogenic locus Notch protein-like [Triticum dicoccoides]	1.00E-55	91.67%	TraesCS4B03G0736100	Neurogenic locus notch homolog protein 2	29.2.4	protein.synthesis.elongation	GO:0005515 MF: protein binding
75912439	S045	JK716075	putative prefoldin subunit 3 [Triticum urartu]	9.00E-47	88.64%	TraesCS5B03G0232700	Prefoldin subunit 3	29.6	protein.folding	GO:0006457 BP: protein folding;GO:0016272 CC: prefoldin complex
75912553	S059	JK716089	elongation factor 1-delta 1-like isoform X2 [Triticum dicoccoides]	8.00E-44	95.06%	TraesCS2B03G0473200	Elongation factor 1-beta	29.2.4	protein.synthesis.elongation	GO:0003746 MF: translation elongation factor activity;GO:0005853 CC: eukaryotic translation elongation factor 1 complex;GO:0006414 BP: translational elongation
75912881	S087	JK716117	chlorophyllide a oxygenase, chloroplastic-like [Triticum dicoccoides]	3.00E-98	99.31%	TraesCS3B03G1434900	Chlorophyllide a oxygenase, chloroplastic	29.3.3	protein.targeting.chloroplast	GO:0005506 MF: iron ion binding;GO:0010277 MF: chlorophyllide a oxygenase [overall] activity;GO:0016491 MF: oxidoreductase activity;GO:0016705 MF: oxidoreductase activity, acting on paired donors, with incorporation or reduction of molecular oxygen;GO:0051537 MF: 2 iron, 2 sulfur cluster binding;GO:0055114 BP: oxidation-reduction process
75912991	S097	JK716127	40S ribosomal protein S27 isoform X1 [Triticum dicoccoides]	4.00E-34	98.82%	TraesCS4B03G0187300	40S ribosomal protein S27	29.2.1.2.1.27	protein.synthesis.ribosomal protein.eukaryotic.40S subunit.S27	GO:0003735 MF: structural constituent of ribosome;GO:0005622 CC: intracellular;GO:0005840 CC: ribosome;GO:0006412 BP: translation
75912992	S098	JK716128	RGS domain-containing serine/threonine-protein kinase A-like isoform X1 [Hordeum vulgare]	2.00E-06	97.44%	TraesCS4B03G0300300	Protein kinase family protein, putative, expressed	29.4	protein.postranslational modification	GO:0004672 MF: protein kinase activity;GO:0005524 MF: ATP binding;GO:0006355 BP: regulation of transcription, DNA-templated;GO:0006468 BP: protein phosphorylation
7591	S1	JK716131	40S ribosomal protein S6-like [Triticum dicoccoides]	6.00	97.0	TraesCS2D03G0	40S ribosomal protein S6	29.2.1	protein.synthesis.ribosomal	GO:0003735 MF: structural constituent of ribosome;GO:0005622 CC: intracellular;GO:0005840 CC: ribosome;GO:0006412 BP: translation

1295	01			E-18	1%	356800		.2.1.6	protein.eukaryotic.40S subunit.S6	
75911299	S105	JK716135	translation machinery-associated protein 22-like [Triticum dicoccoides]	6.00E-70	95.73%	TraesCS3B03G0285500	Translation machinery-associated protein 22	29.2.3	protein.synthesis.initiation	GO:0003743 MF: translation initiation factor activity;GO:0006413 BP: translational initiation
75911303	S109	JK716139	60S ribosomal protein L10-1 [Triticum aestivum]	1.00E-33	83.10%	TraesCS1B03G0360700	Ribosomal protein	29.2.1.2.2.10	protein.synthesis.ribosomal protein.eukaryotic.60S subunit.L10	GO:0003735 MF: structural constituent of ribosome;GO:0005622 CC: intracellular;GO:0005840 CC: ribosome;GO:0006412 BP: translation
75911310	S116	JK716146	probable protein phosphatase 2C 44 [Triticum dicoccoides]	2.00E-70	99.07%	TraesCS2B03G1140900	Protein phosphatase 2c, putative	29.4	protein.postranslational modification	GO:0003824 MF: catalytic activity
75911319	S125	JK716155	Protein translation factor SUI1-like protein [Triticum urartu]	5.00E-09	93.33%	TraesCS1D03G0753700	Protein translation factor SUI1-like protein	29.2.3	protein.synthesis.initiation	GO:0003743 MF: translation initiation factor activity;GO:0006413 BP: translational initiation
75911322	S128	JK716158	ribosomal protein S8 [Triticum turgidum]	4.00E-31	100.00%	TraesCS2B03G0703300	30S ribosomal protein S8	29.2.1.2.1.6	protein.synthesis.ribosomal protein.eukaryotic.30S subunit.S8	GO:0003735 MF: structural constituent of ribosome;GO:0005840 CC: ribosome;GO:0006412 BP: translation
75911324	S130	JK716160	40S ribosomal protein S21 (LOC119285655), mRNA [Triticum dicoccoides]	8.00E-49	90.57%	TraesCS4D03G0415800	40S ribosomal protein S21	29.2.1.2.1.21	protein.synthesis.ribosomal protein.eukaryotic.40S subunit.S21	GO:0003735 MF: structural constituent of ribosome;GO:0005622 CC: intracellular;GO:0005840 CC: ribosome;GO:0006412 BP: translation
75911326	S132	JK716162	vacuolar-processing enzyme-like [Triticum dicoccoides]	8.00E-63	99.28%	TraesCS7A03G1377900	receptor kinase 1	29.4	protein.postranslational modification	GO:0004672 MF: protein kinase activity;GO:0005515 MF: protein binding;GO:0005524 MF: ATP binding;GO:0006468 BP: protein phosphorylation

75911327	S133	JK716163	5.8S ribosomal RNA (LOC119351432), rRNA[Triticum dicoccoides]	1.00E-61	100.00%	TraesCS4D03G0325400	Kinase family protein			GO:0004672 MF: protein kinase activity;GO:0005524 MF: ATP binding;GO:0006468 BP: protein phosphorylation
	Cell stress response and defence									
75911210	S016	JK716046	S-adenosylmethionine decarboxylase [Triticum dicoccoides]	1.00E-14	100.00%	TraesCS2A03G0869700	S-adenosylmethionine decarboxylase proenzyme	22.1.2	polyamine metabolism.synthesis.SAM decarboxylase	GO:0004014 MF: adenosylmethionine decarboxylase activity;GO:0006597 BP: spermine biosynthetic process;GO:0008295 BP: spermidine biosynthetic process
75911215	S021	JK716051	heat shock factor-binding protein [Triticum dicoccoides]	1.00E-49	100.00%	TraesCS7D03G0474400	Heat shock factor-binding protein 1	20.2.1	stress.abiotic.heat	GO:0003714 MF: transcription corepressor activity
75911249	S055	JK716085	putative BPI/LBP family protein At1g04970 isoform X1 [Triticum dicoccoides]	3.00E-81	93.53%	TraesCS5B03G0674300	Lipid-binding serum glycoprotein	35.2	not assigned.unknown	GO:0005615 CC: extracellular space;GO:0008289 MF: lipid binding
75911251	S057	JK716087	heat shock protein 90-5, chloroplastic-like [Triticum dicoccoides]	3.00E-106	97.11%	TraesCS5D03G0595900	Chaperone protein htpG family protein	20.2.1	stress.abiotic.heat	GO:0005524 MF: ATP binding;GO:0006457 BP: protein folding;GO:0006950 BP: response to stress;GO:0051082 MF: unfolded protein binding
75911256	S062	JK716092	unnamed protein product [Triticum turgidum]	2.00E-117	98.19%	TraesCS1A03G0927400	2-oxoglutarate (2OG) and Fe(II)-dependent oxygenase superfamily protein	35.2	not assigned.unknown	GO:0016491 MF: oxidoreductase activity;GO:0055114 BP: oxidation-reduction process
75911258	S064	JK716094	UV-B-induced protein At3g17800, chloroplastic-like [Triticum aestivum]	5.00E-74	97.66%	TraesCS2D03G0994900	UV-B-induced protein, chloroplastic	20.2	stress.abiotic	Unknown

75911262	S068	JK716098	unnamed protein product [Triticum turgidum]	8.00E-75	100.00%	TraesCS6B03G0971900	Superoxide dismutase [Cu-Zn]			Unknown
75911266	S072	JK716102	cytochrome B5-like protein [Triticum aestivum]	9.00E-136	9.44E-01	TraesCS5D03G0453700	Cytochrome B5-like protein	21.2	redox.ascorbate and glutathione	GO:0020037 MF: heme binding
75911268	S074	JK716104	beta purothionin [Triticum urartu]	5.00E-26	60.24%	TraesCS1D03G0943800	Beta purothionin	20.2	stress.abiotic	GO:0006952 BP: defense response
75911269	S075	JK716105	unnamed protein product [Triticum turgidum]	1.00E-36	100.00%	TraesCS6B03G0971900	Superoxide dismutase [Cu-Zn]			Unknown
75911274	S080	JK716110	wPR4a gene [Triticum aestivum]	2.00E-34	83.60%	TraesCS3D03G1160600	Pathogenesis-related protein PR-4	20.1	stress.biotic	GO:0042742 BP: defense response to bacterium;GO:0050832 BP: defense response to fungus
75911278	S084	JK716114	quinolinate synthase, chloroplastic-like isoform X1 [Triticum dicoccoides]	1.00E-149	98.17%	TraesCS5A03G0298000	Quinolinate synthase A	35.2	not assigned.unknown	GO:0008987 MF: quinolinate synthetase A activity;GO:0009435 BP: NAD biosynthetic process;GO:0051539 MF: 4 iron, 4 sulfur cluster binding
75911308	S114	JK716144	cytochrome b5-like [Triticum dicoccoides]	2.00E-50	97.56%	TraesCS3D03G1169900	Cytochrome b5	21.2	redox.ascorbate and glutathione	GO:0020037 MF: heme binding

75911309	S115	JK716145	LMBR1 domain-containing protein 2 homolog A-like [Triticum dicoccoides]	8.00E-137	96.68%	TraesCS5B03G0759900	Disease resistance protein (NBS-LRR class) family			GO:0043531 MF: ADP binding
75911313	S119	JK716149	vulgare mRNA for predicted protein[Hordeum vulgare]	2.00E-146	98.67%	TraesCS5B03G0838800	Thioredoxin superfamily protein	21.1	redox.thioredoxin	Unknown
75911314	S120	JK716150	thioredoxin H4-2-like [Triticum dicoccoides]	1.00E-63	95.92%	TraesCS1B03G0360300	Thioredoxin-like protein	21.1	redox.thioredoxin	GO:0045454 BP: cell redox homeostasis
75911318	S124	JK716154	vulgare acetolactate synthase 1, chloroplastic [Hordeum vulgare]	4.00E-57	97.10%	TraesCS3B03G1388800	Disease resistance protein (NBS-LRR class) family			GO:0043531 MF: ADP binding
75911320	S126	JK716156	calcineurin B-like protein 1 [Triticum dicoccoides]	6.00E-85	100.00%	TraesCS5B03G0063500	F-box protein			GO:0005515 MF: protein binding
75911325	S131	JK716161	monodehydroascorbate reductase 2, peroxisomal-like [Triticum dicoccoides]	4.00E-26	100.00%	TraesCS6A03G0728900	Monodehydroascorbate reductase	21.2	redox.ascorbate and glutathione	GO:0016491 MF: oxidoreductase activity;GO:0050660 MF: flavin adenine dinucleotide binding;GO:0055114 BP: oxidation-reduction process
75911328	S134	JK716164	Bsi1 gene for putative protease inhibitor[Hordeum vulgare]	1.00E-18	83.33%	TraesCS4A03G1248100	ARM repeat superfamily protein			GO:0005488 MF: binding
Signal transduction										

75911212	S018	JK716048	ran-binding protein 1 homolog c-like [Triticum dicoccoides]	3.00E-26	100.00%	TraesCS1B03G0678100	Ran-binding protein 1	30.5	signalling.G-proteins	GO:0046907 BP: intracellular transport
75911225	S031	JK716061	serine/threonine protein phosphatase 2A 55 kDa regulatory subunit B beta isoform [Triticum aestivum]	1.00E-47	91.78%	TraesCS3B03G1464700	Late embryogenesis abundant protein	33.2	development.late embryogenesis abundant	Unknown
75911229	S035	JK716065	probable pyridoxal 5'-phosphate synthase subunit PDX2 [Triticum dicoccoides]	2.00E-19	98.18%	TraesCS6B03G0290800	Pyridoxal 5'-phosphate synthase subunit PdxT	30.11	signalling.light	GO:0004359 MF: glutaminase activity;GO:0042819 BP: vitamin B6 biosynthetic process;GO:0042823 BP: pyridoxal phosphate biosynthetic process
75911234	S040	JK716070	aspartate aminotransferase, chloroplastic-like isoform X2 [Triticum dicoccoides]	2.00E-75	96.55%	TraesCS6D03G0797000	Aspartate aminotransferase	30.3	signalling.calcium	GO:0003824 MF: catalytic activity;GO:0006520 BP: cellular amino acid metabolic process;GO:0008483 MF: transaminase activity;GO:0009058 BP: biosynthetic process;GO:0030170 MF: pyridoxal phosphate binding
75911238	S044	JK716074	lipoxygenase 2.1, chloroplastic-like [Triticum dicoccoides]	8.00E-51	87.76%	TraesCS5D03G0028900	Lipoxygenase	17.7.1.2	hormone metabolism.jasmonate.synthesis-degradation.lipoxygenase	GO:0005515 MF: protein binding;GO:0016491 MF: oxidoreductase activity;GO:0016702 MF: oxidoreductase activity, acting on single donors with incorporation of molecular oxygen, incorporation of two atoms of oxygen;GO:0046872 MF: metal ion binding;GO:0055114 BP: oxidation-reduction process
75911243	S049	JK716079	calcium-dependent protein kinase [Arachis hypogaea]	2.00E-27	93.33%	TraesCS2B03G0142700	2-oxoglutarate (2OG) and Fe(II)-dependent oxygenase superfamily protein	17.5.1	hormone metabolism.ethylene.synthesis-degradation	GO:0016491 MF: oxidoreductase activity;GO:0055114 BP: oxidation-reduction process
75911260	S066	JK716096	transcript variant X1, mRNA[Triticum dicoccoides]	2.00E-173	96.56%	TraesCS5B03G1336100	Transducin/WD-like repeat-protein	33.99	development.unspecified	GO:0005515 MF: protein binding
75911267	S073	JK716103	Programmed cell death protein 4 [Triticum urartu]	1.00E-55	90.4	TraesCS2B03G0	Programmed cell death protein 4	28.99	DNA.unspecified	GO:0005488 MF: binding

					8%	928300				
75911272	S078	JK716108	zeaxanthin epoxidase, chloroplastic-like [Triticum dicoccoides]	1.00E-39	100.00%	TraesCS2B03G0871900	Zeaxanthin epoxidase, chloroplastic	17.1.1.1.1	hormone metabolism.abscisic acid.synthesis-degradation.synthesis.zeaxanthin epoxidase	GO:0005515 MF: protein binding;GO:0009507 CC: chloroplast;GO:0009540 MF: zeaxanthin epoxidase [overall] activity;GO:0009688 BP: abscisic acid biosynthetic process;GO:0016020 CC: membrane;GO:0016491 MF: oxidoreductase activity;GO:0055114 BP: oxidation-reduction process;GO:0071949 MF: FAD binding
75912879	S085	JK716115	unnamed protein product [Triticum turgidum]	2.00E-103	98.84%	TraesCS3A03G0507700	Evolutionarily conserved C-terminal region 2	30.3	signalling.calcium	GO:0003723 MF: RNA binding
75912988	S094	JK716124	multiprotein-bridging factor 1a-like [Hordeum vulgare]	6.00E-95	98.59%	TraesCS2A03G0939700	Multiprotein-bridging factor, putative	17.5.3	hormone metabolism.ethylene.induced-regulated-responsive-activated	GO:0003677 MF: DNA binding;GO:0043565 MF: sequence-specific DNA binding
75911305	S111	JK716141	IAA-amino acid hydrolase ILR1-like 7 [Triticum dicoccoides]	1.00E-76	96.72%	TraesCS5A03G0253100	IAA-amino acid hydrolase ILR1	17.2.1	hormone metabolism.auxin.synthesis-degradation	GO:0008152 BP: metabolic process;GO:0016787 MF: hydrolase activity
75911217	S123	JK716153	CBL-interacting protein kinase 8[Triticum dicoccoides]	5.00E-57	88.50%	TraesCSU03G0060600	Gluconokinase	35.2	not assigned.unknown	GO:0005975 BP: carbohydrate metabolic process;GO:0016301 MF: kinase activity
Transcription										
75911206	S012	JK716042	MADS-box transcription factor 51-like isoform X2 [Triticum dicoccoides]	3.00E-31	89.04%	TraesCS4D03G0803000	MADS-box transcription factor	27.3.24	RNA.regulation of transcription.MADS box transcription factor family	GO:0003677 MF: DNA binding;GO:0046983 MF: protein dimerization activity

75911254	S060	JK716090	NAC domain-containing protein 41-like [Triticum dicoccoides]	2.00E-61	79.14%	TraesCS7B03G0534600	multimeric translocon complex in the outer envelope membrane 132			Unknown
75911270	S076	JK716106	chromatin structure-remodeling complex protein SYD-like [Triticum turgidum]	7.00E-121	100.00%	TraesCS7A03G0477200	Chromatin remodeling protein-like			GO:0005524 MF: ATP binding;GO:0042393 MF: histone binding
75911275	S081	JK716111	2-hydroxy-palmitic acid dioxygenase MPO1-like [Triticum dicoccoides]	2.00E-84	99.44%	TraesCS7A03G0985500	YGL010w-like protein	27.3.11	RNA.regulation of transcription.C2H2 zinc finger family	Unknown
75911283	S089	JK716119	Uncharacterized RNA-binding protein C23E6.01c [Triticum urartu]	1.00E-72	99.24%	TraesCS2A03G0475600	RNA-binding family protein	27.3.99	RNA.regulation of transcription.unclassified	GO:0003676 MF: nucleic acid binding
75911293	S099	JK716129	chloroplast stem-loop binding protein of 41 kDa b, chloroplastic-like [Triticum dicoccoides]	3.00E-42	97.40%	TraesCS5D03G0111300	Chloroplast stem-loop-binding protein	27.3.99	RNA.regulation of transcription.unclassified	GO:0003824 MF: catalytic activity;GO:0050662 MF: coenzyme binding
75911307	S113	JK716143	U2 small nuclear ribonucleoprotein B"-like [Triticum dicoccoides]	4.00E-62	98.29%	TraesCS4B03G0531900	Small nuclear ribonucleoprotein	27.1.1	RNA.processing.splicing	GO:0003676 MF: nucleic acid binding
75911323	S129	JK716159	ubiquitin-conjugating enzyme E2 28-like isoform X1 [Panicum virgatum]	7.00E-05	95.24%	TraesCS7B03G0667900	Ubiquitin-conjugating enzyme E2	27.3.11	RNA.regulation of transcription.C2H2 zinc finger family	Unknown
Transport										

75911200	S005	JK716036	conserved hypothetical protein [Talaromyces stipitatus]	1.00E-15	67.80%	TraesCS3D03G0906500	ATP-binding ABC transporter	34.16	transport.ABC transporters and multidrug resistance systems	GO:0005524 MF: ATP binding;GO:0006810 BP: transport;GO:0016021 CC: integral component of membrane;GO:0016887 MF: ATPase activity;GO:0042626 MF: ATPase activity, coupled to transmembrane movement of substances;GO:0055085 BP: transmembrane transport
75911214	S020	JK716050	non-specific lipid-transfer protein Cw18 [Triticum dicoccoides]	1.00E-07	83.33%	TraesCSU03G0342400	Non-specific lipid-transfer protein	11.10.2	lipid metabolism.glycolipid synthesis.DGDG synthase	GO:0006869 BP: lipid transport;GO:0008289 MF: lipid binding
75911216	S022	JK716052	type 1 non-specific lipid transfer protein precursor [Triticum turgidum]	5.00E-11	80.70%	TraesCSU03G0129800	Non-specific lipid-transfer protein	11.10.2	lipid metabolism.glycolipid synthesis.DGDG synthase	GO:0006869 BP: lipid transport;GO:0008289 MF: lipid binding
75911226	S032	JK716062	putative lipid-transfer protein DIR1 [Triticum dicoccoides]	2.00E-43	92.38%	TraesCSU03G0269700	Protease inhibitor/seed storage/lipid transfer protein family protein	11.10.2	lipid metabolism.glycolipid synthesis.DGDG synthase	GO:0006869 BP: lipid transport;GO:0008289 MF: lipid binding
75911233	S039	JK716069	probable calcium-transporting ATPase 9, plasma membrane-type isoform X2 [Triticum aestivum]	7.00E-97	86.36%	TraesCS5B03G0374700	Calcium-transporting ATPase	34.3	transport.amino acids	GO:0000166 MF: nucleotide binding;GO:0005388 MF: calcium-transporting ATPase activity;GO:0005516 MF: calmodulin binding;GO:0005524 MF: ATP binding;GO:0016020 CC: membrane;GO:0016021 CC: integral component of membrane;GO:0046872 MF: metal ion binding;GO:0070588 BP: calcium ion transmembrane transport
75911235	S041	JK716071	nascent polypeptide-associated complex subunit alpha-like protein 1 [Triticum dicoccoides]	2.00E-51	88.82%	TraesCS1B03G0701500	Nascent polypeptide-associated complex subunit alpha-like protein	28.1	DNA.synthesis/chromatin structure	Unknown
75911257	S063	JK716093	probable anion transporter 5, chloroplastic [Triticum dicoccoides]	2.00E-73	98.45%	TraesCS5A03G0859000	Phosphate transporter	34.99	transport.misc	GO:0016021 CC: integral component of membrane;GO:0055085 BP: transmembrane transport
75911259	S065	JK716095	phosphoribulokinase, chloroplastic [Triticum dicoccoides]	9.00E-53	93.83%	TraesCS6B03G0849400	Phosphoribulokinase	34.99	transport.misc	GO:0005524 MF: ATP binding;GO:0005975 BP: carbohydrate metabolic process;GO:0008152 BP: metabolic process;GO:0008974 MF: phosphoribulokinase activity;GO:0016301 MF: kinase activity

75911282	S088	JK716118	thylakoid membrane protein slr0575-like isoform X1 [Triticum dicoccoides]	2.00E-66	96.67%	TraesCS7A03G0701100	acclimation of photosynthesis to environment	31.4	cell.vesicle transport	Unknown
75911289	S095	JK716125	protein detoxification 21-like [Triticum dicoccoides]	4.00E-95	98.20%	TraesCS5A03G0396000	Protein DETOXIFICATION	34.99	transport.misc	GO:0006855 BP: drug transmembrane transport;GO:0015238 MF: drug transmembrane transporter activity;GO:0015297 MF: antiporter activity;GO:0016020 CC: membrane;GO:0055085 BP: transmembrane transport
75911302	S108	JK716138	clathrin interactor EPSIN 1-like [Triticum dicoccoides]	5.00E-72	88.24%	TraesCS2D03G0983900	Clathrin interactor EPSIN 1	35.2	not assigned.unknown	Unknown
75911316	S122	JK716152	probable V-type proton ATPase subunitd [Triticum dicoccoides]	2.00E-39	87.50%	TraesCS3B03G0628900	V-type proton ATPase subunit d	34.1.1	transport.p- and v-ATPases.H+-transporting two-sector ATPase	GO:0015078 MF: hydrogen ion transmembrane transporter activity;GO:0015991 BP: ATP hydrolysis coupled proton transport;GO:0033179 CC: proton-transporting V-type ATPase, V0 domain
Unknown										
75911208	S014	JK716044	testis-expressed protein 2-like [Triticum aestivum]	2.00E-110	100.00%	TraesCS6A03G1028700	Testis-expressed sequence 2 protein			Unknown
75911211	S017	JK716047	calphotin-like [Rhopalosiphum maidis]	7.00E-11	97.06%	TraesCS2D03G0059300	ATP sulfurylase (Sulfate adenylyltransferase)	14.1	S-assimilation.ATPS	GO:0000103 BP: sulfate assimilation;GO:0004781 MF: sulfate adenylyltransferase (ATP) activity
75911219	S025	JK716055	protein LURP-one-related 15-like [Triticum dicoccoides]	2.00E-51	90.17%	TraesCS1D03G0764300	Endonuclease 8-like 3			GO:0008270 MF: zinc ion binding

75911237	S043	JK716073	protein unc-13 homolog isoform X2 [Triticum dicoccoides]	1.00E-101	98.78%	TraesCS1A03G0128600	Mammalian uncoordinated homology 13, domain 2	35.2	not assigned.unknown	Unknown
75912506	S056	JK716086	ubiquitin-associated protein [Triticum aestivum]	6.00E-132	94.97%	TraesCS6B03G0686900	Ubiquitin-associated protein	35.2	not assigned.unknown	GO:0005515 MF: protein binding;GO:0008270 MF: zinc ion binding
75912764	S070	JK716100	unnamed protein product [Triticum turgidum]	6.00E-21	100.00%	TraesCS4B03G0678900	Nodulin-related protein 1, putative	35.2	not assigned.unknown	Unknown
75912096	S102	JK716132	unnamed protein product [Triticum turgidum]	8.00E-94	100.00%	TraesCS6B03G1051000	RING finger protein			Unknown
75912098	S104	JK716134	UPF0047 protein YjbQ-like [Triticum dicoccoides]	1.00E-29	96.36%	TraesCS7D03G1216200	UPF0047 protein YjbQ			Unknown
75913111	S117	JK716147	vulgare SUMO-conjugating enzyme SCE1-like [Hordeum vulgare]	2.00E-123	98.45%	TraesCS5A03G0040700LC	Unknown			Unknown
75913221	S127	JK716157	protein COFACTOR ASSEMBLY OF COMPLEX C SUBUNIT B CCB1, chloroplastic-like [Triticum dicoccoides]	2.00E-04	50.94%	TraesCS3B03G1346500	Cofactor assembly of complex C	35.2	not assigned.unknown	Unknown

Supplementary Table S2. List RT-PCR primers designed for the analysised wheat genes

No.	User_Id	dbEST_Id	Nr annnotation/	Sequence of Primer	
Q1	S052	JK716082	Chlorophyll a-b binding protein 1C, chloroplastic	F: TTGAGAACCTTGCCGACCAC	R: CCACACTCCCGTTACCTTGG
Q2	S069	JK716099	protein THYLAKOID FORMATION1, chloroplastic	F: CCTGAGCAATACAGGGCTGA	R: GAACCGGCTGTAGCTGAAGT
Q3	S009	JK716040	psbP-like protein 1, chloroplastic	F: TTCGTCTACCCGTTCGGATG	R: TGTTGATGCTGACGCTCTCC
Q4	S013	JK716043	light-harvesting complex-like protein OHP2, chloroplastic	F: CGCCGTGCATTTGTATGGTC	R: TCCAAGACGCTGGTCTCTAAC
Q5	S046	JK716076	fructose-1,6-bisphosphatase, cytosolic-like	F: TTCCGTTCGGGCTCTTCT	R: ACTGGGACACGCCTACTGC
Q6	S036	JK716066	ribulose-1,5-bisphosphate carboxylase/oxygenase large subunit	F: ACGTGCTCTACGTTTGGAGG	R: CACGTAGACACTCATAACACGC
Q7	S079	JK716109	phospholipase A1-II 7-like	F: CCGCTATGCTGACGAAGAGT	R: TACCTCCTCGAGAACCTGCT
Q8	S050	JK716080	protein CHUP1, chloroplastic-like	F: AGTGGAGCAGAGTGTTTACG	R: GCCCTGGAGAAGCAAGAA
Q9	S100	JK716130	alpha-L-arabinofuranosidase 1-like isoform X2	F: CTTGGTTCACTAGCAGAGGC	R: GTGGCGCATAACTTGCCATC
Q10	S030	JK716060	malate dehydrogenase, cytoplasmic-like	F: ACTTGTTCCTTTGTTGACCAGG	R: GTTGCAACAACTCCCTTGAGA
Q11	S038	JK716068	glyceraldehyde-3-phosphate dehydrogenase 1, cytosolic-like	F: ACGTCGAGGAGGATTTGGTC	R: TGCTGTAACCCCACTCGTTG
Q12	S116	JK716146	probable protein phosphatase 2C 44	F: ATGAAAGGCAGAGGATTGAA	R: GTGCGTCTTGAGGCTATGGT
Q13	S109	JK716139	60S ribosomal protein L10-1	F: TCTTGAGGCTGCCCGTATTG	R: AAGCACCCCTCATACCAGTC

Q14	S125	JK716155	Protein translation factor SUI1-like protein	F: GGAGCTAGGCCAGGTCATTC	R: GCAAGACACGGTTCGACAAG
Q15	S105	JK716135	translation machinery-associated protein 22-like	F: GAAGAGGTGAAACGACTGCC	R: CACCACAGTAACACACTTGCG
Q16	S087	JK716117	chlorophyllide a oxygenase, chloroplastic-like	F: ACTTCGCACCCTGGATGAAG	R: GACTGCATCCCTCCATAGCC
Q17	S039	JK716069	probable calcium-transporting ATPase 9, plasma membrane-type isoform X2	F: TAAGCGCGACGGTGGTATTC	R: CGCTCACCAACCAGAATTGC
Q18	S063	JK716093	probable anion transporter 5, chloroplastic	F: CTGGGTCGTTGTTGCTTTCC	R: ACACCAACGATTCCAGGCAC
Q19	S121	JK716151	probable aquaporin PIP2-2	F: TTCTCCACCAAGGACTACTCG	R: TAGAGGAAGAGGAGCGTGG
Q20	S012	JK716042	MADS-box transcription factor 51-like isoform X2	F: GTGCAAGCACCAACAATGGG	R: CGTTGCGCCAACATCTTCTTG
Q21	S060	JK716090	NAC domain-containing protein 41-like	F: TCGTAGGTGCCCTGTTTCTTC	R: CACTGGAACTGCCTCCCATC
Q22	S044	JK716074	lipoxygenase 2.1, chloroplastic-like	F: GACCTCTTGTCCACGCACTC	R: CGTTGTTCCACTCATCCACC
Q23	S018	JK716048	ran-binding protein 1 homolog c-like	F: TCTCGACCACGAAGATGCAGG	R: AGCTCTCCGTCAGCAAAGTC
Q24	S031	JK716061	serine/threonine protein phosphatase 2A 55 kDa regulatory subunit B beta isoform	F: GAGTTGAGTCACACCTGCAAAC	R: ACGGTGAAACATCTGTGGAC
Q25	S094	JK716124	multiprotein-bridging factor 1a-like	F: GGCACAACTTGCTCAGATGAT	R: ACTTCAAGGCATTACTTCTTGCT
Q26	S016	JK716046	S-adenosylmethionine decarboxylase	F: TACTATGCCACCGAGCAACC	R: AAGTAGCGGTTCAGGACATC
Q27	S131	JK716161	monodehydroascorbate reductase 2, peroxisomal-like	F: GGCACGTCTTTCTTGCTTTC	R: CGTTATCGGTGGTTGTCTGG
Q28	S134	JK716164	Bsi1 gene for putative protease inhibitor	F: ACGTGAACGCCGTCTATTTC	R: CCACCAACGCACAATCATTA

Q29	S055	JK716085	putative BPI/LBP family protein At1g04970 isoform X1	F: TTTCTGGGAATAATCTTGTTGG	R: TGATGACGAAGCCCTTGATG
Q30	S080	JK716110	wPR4a gene	F: GTAATCTAGTCGCGGCAGTC	R: ACCAAGATCGACACCAACGG
